# Understanding the omicron variant (B.1.1.529) of SARS-CoV-2: Mutational impacts, concerns, and the possible solutions

**DOI:** 10.1016/j.amsu.2022.103737

**Published:** 2022-05-07

**Authors:** Fahadul Islam, Manish Dhawan, Mohamed H. Nafady, Talha Bin Emran, Saikat Mitra, Om Prakash Choudhary, Aklima Akter

**Affiliations:** a Department of Pharmacy, Faculty of Allied Health Sciences, Daffodil International University, Dhaka 1207, Bangladesh; b Department of Microbiology, Punjab Agricultural University, Ludhiana,141004, Punjab, India; c Trafford College, Altrincham, Manchester, WA14 5PQ, UK; d Faculty of Applied Health Science Technology, Misr University for Science and Technology, Giza 12568, Egypt; e Department of Pharmacy, BGC Trust University Bangladesh, Chittagong 4381, Bangladesh; f Department of Pharmacy, Faculty of Pharmacy, University of Dhaka, Dhaka 1000, Bangladesh; g Department of Veterinary Anatomy and Histology, College of Veterinary Sciences and Animal Husbandry, Central Agricultural University (I), Selesih, Aizawl, India

**Keywords:** Variant of concern (VOC), Omicron variant (B.1.1.529), SARS-CoV-2, COVID-19

## Abstract

Despite many nations' best efforts to contain the so-called COVID-19 pandemic, the emergence of the SARS-CoV-2 Omicron strain (B.1.1.529) has been identified as a serious concern. After more than two years of COVID-19 pandemic and more than a year of worldwide vaccination efforts, the globe will not be free of COVID-19 variants such as Delta and Omicron variants. According to current statistics, the Omicron variant has more than 30 mutations when contrasted to other VOCs such as Alpha (B.1.1.7), Beta (B.1.351), and Delta (B.1.617.2). High numbers of changes, particularly in the spike protein (S-Protein), raise worries about the virus's capacity to resist pre-existing immunity acquired by vaccination or spontaneous infection and antibody-based therapy. The Omicron variant raised international concerns, resuming travel bans and coming up with many questions about its severity, transmissibility, testing, detection, and vaccines efficiency against it.

Additionally, inadequate health care infrastructures and many immunocompromised individuals increase the infection susceptibility. The current status of low vaccination rates will play a significant role in omicron spreading and create a fertile ground for producing new variants. As a result, this article emphasizes the mutational changes and their consequences. In addition, the potential preventing measures have been examined in detail.

## Highlights

• In South Africa, WHO designated the first cases of the SARS-CoV-2 Omicron variant (B.1.1.529).
• This variation is notable for having an extremely high number of spike protein mutations.
• Omicron has about 50 mutations, particularly 32 spike protein mutations.
• Omicron considers the most common variant in South Africa as an alternative to Delta.

## 1 Introduction

Multiple variants of SARS-CoV-2 have been identified since the start of the COVID-19 pandemic. These variants have been related to a significant increase in fatality rates in several countries [1,2]. The World Health Organization (WHO) has previously identified five VOCs: Alpha, Beta, Gamma, Delta, and Omicron variants. The emergence of novel SARS-CoV-2 variants, notably VOCs like Delta, Beta, and Alpha, has been linked to the rapid increase of COVID-19 cases simultaneously among several nations [2,3]. The Omicron variant of SARS-CoV-2 is a highly modified strain that has quickly spread worldwide and competed with other VOCs [4]. In early November, Omicron was found in Botswana. On November 24, 2021, South Africa notified the WHO, and on November 26, 2021, it was classified as a VOC. Omicron variant has a substantial percentage of previously described mutations in other VOCs, along with novel mutations, including at least 32 mutations in the spike protein (S- protein) alone, compared to 16 alterations in the already highly transmissible delta variant, as well as other viral replication proteins including NSP12 and NSP14 [[5], [6], [7], [8]].

Many mutations (50 mutations) found in the Omicron variant have sparked widespread alarm among scientists [9]. Omicron contains specific distinctive changes compared to other VOCs [10], mainly in the Spike protein (S-protein), which has been linked to its higher transmissibility even among vaccinated people [11,12]. In comparison to other VOCs like Alpha (B.1.1.7), Beta (B.1.351), and Delta (B.1.617.2), the Omicron variant (B.1.1.529) has more than 30 mutations, according to current statistics [4,8,12]. As a result, researchers were apprehensive about the Omicron variant's higher mutation frequency. Several scientists expressed concerns over the past months, including enhanced transmissibility, reduced vaccination efficiency, and an increased risk of reinfection [4,8,12].

As a result, we will focus on various features of the Omicron variant in this article to better comprehend its consequences and concerns in numerous nations' significant attempts to mitigate the devastating effects of the COVID-19 pandemic.

## 2 Mutations in the omicron variant

Among five major VOCs reported, the Omicron variant is significantly mutated [[13], [14], [15]]. The Omicron variant has roughly 50 mutations across its genome, with almost 32 mutations in the S-protein coding [12,16]. Modifications on the S- protein includes A67V, Δ69–70, T95I, G142D/Δ143-145, Δ211/L212I, ins214EPE, G339D, S371L, S373P, S375F, K417 N, N440K, G446S, S477 N, T478K, E484A, Q493K, G496S, Q498R, N501Y, Y505H, T547K, D614G, H655Y, N679K, P681H, N764K, D796Y, N856K, Q954H, N969K, L981F [12] (Figure No 1)**.** T478K, N501Y, N655Y, N679K, and P681H are the mutations that overlap with other VOCs and VOIs like Delta, Gamma, Alpha, and Beta [17,18]. However, the omicron has been described as a highly altered version with a “unique constellation of mutations [12]. As a result, it's critical to understand the Omicron variant's mutation landscape. Specifically, the influence of mutations on antibody escape is described for SARS-CoV-2 variants [12,18].

**Fig. 1:**
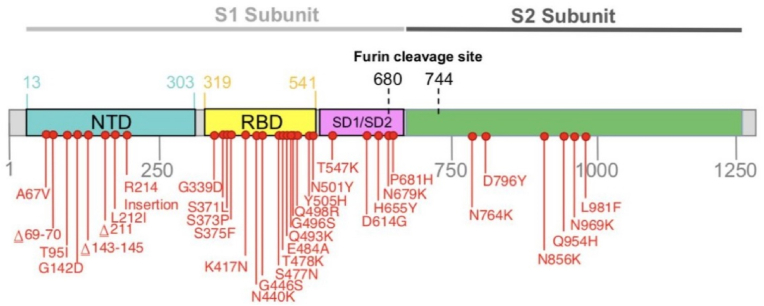
Representation of mutations on the spike gene of the Omicron variant (BA.1 lineage). Many mutations in the S-protein, particularly in the RBD of S-protein, lead to the enhanced interaction with ACE2 receptors. The enhanced binding properties of RBD with ACE2 have been postulated as a critical reason for the increased transmissibility of the Omicron variant [19]. Abbreviations: S-protein (Spike protein), RBD (Receptor Binding Domain), ACE2 (Angiotensin Converting Enzyme 2) (Source: https://www.who.int/).

Mutations in the RBD (receptor-binding domain) and NTD (N-terminal domain) have recently been discovered to substantially influence the disease's infectiousness and severity. Antibody escape may be caused by V445E and K444 Q/R/N in the RBD. In addition, K150 T/Q/R/E and N148S mutations in the NTD have been postulated to influence the vaccine effectiveness [12,20]. Another study found that the Omicron variant reduced neutralization ability after two doses of the Oxford–AstraZeneca vaccine or two doses of the Pfizer–BioNTech vaccine [20], indicating a nAbs (neutralizing antibodies) escape event [12,20,21]. Moreover, as shown in the Alpha (P681H) and Gamma (P681H), three alterations across the furin cleavage site may improve transmissibility and replication17 (H655Y, N679K) [22]. The nsp6 deletion Δ105-107 (also observed in Alpha, Beta, and Gamma VOCs) may be linked to other escape of host defense and increased transmissibility outside the spike protein [23]. R203K and G204R nucleocapsid alterations (also observed in Alpha and Gamma VOCs) may be linked to higher infectiousness [24].

Bhattacharya et al. (2022) [12] evaluated mutations in antibody-binding areas and found a few key mutations that closely matched earlier mutations, particularly N501Y, D614G, H655Y N679K, and P681H. Q493K, G496S, Q498R, S477 N, G466S, N440K, and Y505H are novel variations discovered in RBD. Additional alterations were found in the NTD (Δ143–145, A67V, T95I, L212I, and 211), including one in the fusion peptide (D796Y). K417 N, E484A, Q493K, Q498R, N501Y, and Y505H are among the mutations in the antibody-binding area and those near the antibody-binding region (S477 N, T478K, G496S, G446S, and N440K). Mutations in areas critical for the binding between spike proteins and neutralizing antibodies were studied, and it has been postulated that the changes can influence the neutralization capabilities **(**Table 1**)**. In addition, they also looked at how critical antibody-binding mutations such as K417 N, T478K, E484A, and N501Y influenced antibody affinity, ACE2 association stability, and the potential of amino acid replacement. The antibody-binding affinity is destabilized by all four alterations [12].

**Table 1:** The major mutations in the S-protein which has been postulated as key mutations to raise the infectiousness and transmissibility.

S. No.	Mutations in the S-protein	Impact of the mutation on transmissibility and infection rate	Other noticeable impacts	References
	G339D	Increase the binding affinity of S-protein with ACE2 receptor	–	[25]
	S373P	Increase in the infection rate	–	[26]
	N440K	Increase in the infection	–	[27]
	G446	Increase in the infection	–	[28]
	S477 N	Increase the binding affinity of S-protein with ACE2 receptor	S477 N mutation was found to increase the resistance to the neutralization by human convalescent plasma (CP) but is susceptible to vaccine-induced sera	[29,30]
	T478K	Increase in the infectiousness capacity	Increase in resistance to the convalescent sera	[26,29]
	Q493R	Increase in infection rate	–	[30]
	G496S	Increase in infection rate	–	[26]
	N501Y	Increase the binding affinity of S-protein with ACE2 receptor	–	[25]
	Y505H	Increase in the infectiousness	–	[28]
	D614G	Increase in infectiousness and transmissibility	Lower Ct values were observed in G614 infections indicating higher viral load	[25,[31], [32], [33], [34]]
	H655Y	Increase in transmissibility	–	[35]

RNA polymerase (Nsp12) and nonstructural protein 14 (Nsp14) are required for viral reproduction, although it is unknown if changes in these portions of omicron contribute to more significant genetic changes. Omicron also possesses nucleocapsid protein alterations such as R203K and G204R, which are not specific to omicron but are connected to enhanced sub-genomic RNA production and viral multiplication [11,12]. It is essential to consider that many mutations are present in the Omicron variant's S-protein and the other regions of the genome, which can be a necessary factor for their enhanced transmissibility. Hence, further research is needed to understand immunological and antibody escape [12].

## 3 Impact on transmissibility and severity

The effectiveness with which the Omicron variant may transfer from one person to another has yet to be determined. Not only in Africa but all around the world, the Omicron form became the most common. In South Africa, the fast rise of the Omicron variation over the Delta version has prompted significant concerns that the Omicron variant is more transmissible and infectious than the Delta variant and other VOCs. It was initially questionable if the Omicron variant was much more contagious than other VOCs, particularly the Delta variant, due to the small number of cases in South Africa when Omicron first appeared. Modifications in the S-protein structure imply that the Omicron form of SARS-CoV-2 is more transmissible than the original strain [36]. However, the Omicron variant is substantially more spreadable than the Delta variant, according to multiple recent studies. Yet, the severity of the sickness produced by the Omicron variant is comparable to the Delta form [36].

In a recent investigation, infection rates were four times greater in the Omicron variant than in the wild type of SARS-CoV-2. Furthermore, the Omicron variant displays a considerable increase in infectiousness [37]. A variety of pseudoviruses were compared to the wild-type SARS-CoV-2 using linear regressions. The infection rate of the gamma variant was like that of the wild-type SARS-CoV-2. The infection rate was lower in the Beta variant.

Furthermore, the Delta showed a two-fold boost in target cell infection efficiency. Such findings underline the significance of SARS-CoV-2 S-protein mutations, which considerably impact infectivity. Furthermore, compared to other VOCs, efficient interaction of ACE2 receptors with S-Protein of the Omicron variant has been linked to enhanced infectiousness [37].

According to South Africa's preliminary data, there were no peculiar signs connected with omicron. A few people are asymptomatic, as they are with other variations [38]. According to anecdotal evidence from doctors in South Africa, Omicron generates milder symptoms and less significant sickness. On the other hand, these milder incidences occurred in younger persons [39]. According to early research, omicron is less severe than other forms, with a risk of hospitalization varying between 15% and 80% lesser than the Delta variant [40,41]. Omicron may not cause severe disease, especially in those vaccinated and who have received a booster dose [37]. Most reported cases to fall into the category of clinically asymptomatic or moderate instances. Runny nose, headache, tiredness (mild or severe), sneezing, and sore throat are signs of the Omicron variant [42,43]. On the other hand, the youngsters participated in the Omicron-led fourth wave in South Africa, where early data revealed that the risk of hospital admission for children was 20% greater than in the D614G-led first wave (SAMRC, 2021). In ex vivo culture investigations, Hong Kong University researchers discovered that omicron multiplies 70 times faster than the Delta variant in human bronchus but ten times slow in human lung tissue, which might explain why omicron infected individuals with a milder illness [44].

## 4 Impact on immune response and convalescent plasma

Convalescent plasma (CP) from patients who had previously been infected with ancestral SARS-CoV-2 strains was evaluated in vitro and shown to have much lower neutralization against VOCs like the Beta (B.1.351) variant [45]. Hence, it is essential to assess the efficiency of CP against the Omicron variant. As large unvaccinated groups around the globe continue to raise the possibility of variant development, it is critical to rediscover the potential of CP and CP-based therapy against VOCs, particularly against Beta, Delta, and Omicron variants [46]. According to recent research, the Omicron variation can avoid antibodies generated by the original strain and vaccination [15]. With just two mutations in the RBD, the Delta variant shows a slight reduction in the RBD's binding capacity to both vaccinated and convalescent sera, which is consistent with recent research [15,47]. On the other hand, omicron successfully evades antibodies induced by ancestral variations and inactivated vaccines, despite a significant reduction in the binding potential to its RBD [15,47]. Several recent findings indicate that the omicron variant shows an unprecedented degree of neutralizing antibody escape [48]; they also suggest that boosting and promoting affinity maturation of antibodies in persons who have previously been infected or vaccinated with the use of existing Wuhan-hu-1–based vaccine immunogens will provide additional protection against infection with the omicron variant and subsequent disease [49].

## 5 Concerns over the detection and diagnosis

The Spike protein in omicron has a mutation 69–70 (identical to Alpha but distinct to Delta). Because one PCR test, ThermoFisher TaqPath, can identify the deletion of this target gene (also known as S gene dropout or S gene target malfunction), it could be used as an early indicator to distinguish between Omicron and Delta, awaiting sequenced validation. On omicron, it's unclear how well quick antigen tests will work. Several (but not all) assays on the targeted market the nucleocapsid protein rather than the spike protein; therefore, they should continue to operate. Rapid antigen testing is being studied and see if they are affected [50,51].

Owing to the significant number of alterations in the Omicron variant, concerns have been raised over the performance of commercial and in-house produced SARS-CoV-2 specific PCR tests [52]. Furthermore, as was the case with the Alpha variant, partial detection failure of specific assays may be utilized to diagnose possible Omicron instances.

## 6 Where we stand with the vaccines′ effectiveness

A preprint study posted online by South African researchers found that Omciron could increase the risk level among individuals with immunity acquired through the previous infection more than other variants. Additionally, South Africa's National Institute for Communicable Diseases NICD found that reinfections in South Africa have risen as omicron expands [53]. It's uncertain whether omicron may avoid infection and vaccination immunity, and if so, to what magnitude. Approximately 24% of the population in South Africa, one of the nations where Omicron infections are on the rise, has been entirely vaccinated, and it is unclear how many of the affected individuals have been vaccinated during this period [54]. There have been press reports of B.1.1.529 outbreaks in fully vaccinated travelers in Botswana and Israel. There have also been accounts of 61 out of 624 South African travelers testing positive for COVID-19 on arrival in Amsterdam, such as those infected with omicron. Given that entrance to Amsterdam meets the standard of vaccination or a negative test, these instances are probable recently or despite immunization [55,56].

While several receptor-binding domain (RBD) alterations in omicron's spike protein imply a significant risk of an immunological breakaway from antibody-mediated prophylaxis, immune discharge from memory T cells targeted towards non-surface proteins subsequent vaccination or infection is more challenging to assess. When virus replication results in spike protein changes that elude pre-existing neutralizing antibodies, memory T cell responses might provide a path to long-term protection, this could happen by providing more practical assistance to activated naive B cells reacting to the modified spike protein (CD4 T cells) or by directly lysing SARS-CoV-2 infected cells (CD8 T cells) [57]. Although presently available vaccines might provide safeguards against hospitalization and death, in vitro studies have evaluated the neutralizing potency of vaccinee and convalescent sera against Omicron pseudo- or live virus estranges are expeditiously devoted to understanding the virus's escape prospects against both vaccination and infection-acquired immune function. This information should be available within two to three weeks [58]. Recent research discovered that the omicron variant's vaccine efficacy was lower than the delta variant's at all intervals following immunization and for all introductory courses and booster doses studied [59]. According to the investigators, two doses of BNT162b2 or ChAdOx1 nCoV-19 vaccination are insufficient to protect against infection with the omicron form and moderate illness. More research will be required to measure protection against severe disease and the length of protection following booster immunization [59].

## 7 Vaccine inequity and hesitancy: major concerns amid the omicron emergence

The rise of the Omicron variant and its speedy expansion indicate wealthy nations' critical role in considering the equal distribution of COVID-19 vaccines globally. Inequalities in vaccine availability among countries have been identified as a significant problem, resulting in SARS-CoV-2 variants [60]. Only around a fifth of Africans is fully inoculated against the COVID-19, including millions of healthcare workers and vulnerable communities. There are still unvaccinated vulnerable groups worldwide that need to be vaccinated as soon as possible. Other VOCs may evolve in the future if SARS-COV-2 continues to circulate, particularly among the unvaccinated, and the resultant viral evolution may ultimately result in vaccine-resistant varieties [46]. Without this equality of vaccines, disruption will contribute to prolonging the pandemic, putting the whole world at continued risk of COVID-19 and impacting their economies [46].

Furthermore, vaccine hesitancy is one of the most severe risks to global health because it jeopardizes our ability to abolish infectious diseases by establishing herd immunity among the entire population through vaccination [46]. Those individuals who refuse to be vaccinated or who choose not to receive the COVID-19 vaccine could slow the cumulative vaccination pace and coverage, resulting in lower vaccination rates and obstructing worldwide efforts to curb the dissemination of SARS-CoV-2, as unvaccinated individual people could function as SARS-CoV-2 reservoirs, resulting in more breakouts [46,61].

## 8 Preventing measures

The Omicron variant is developing at a breakneck pace and presents a serious concern to human health. The discovery of the omicron variant serves as a sobering reminder that, while we've made significant progress against COVID-19 in the previous two years, there is still much more work to be done in this area [62]. There is a need for rapid diagnosis, thorough surveillance, and monitoring of the new SARS-CoV-2 variations, essential [[63], [64], [65]]. There is a pressing need to minimize vaccination hesitancy while simultaneously developing even more effective vaccines, which is now underway [66,67]. It is necessary to create a vaccination that is effective enough to reach the world's population. The safety precautions implemented during the early waves of SARS-CoV-2, such as the adoption of quarantine, wearing masks and other sophisticated protective equipment, and excellent hygiene practices, must be followed up continuously [2,15].

One of the primary tasks in any Omicron-stricken country should be to enhance medical facilities and make these upgraded facilities available and accessible to virus-infected patients, particularly those who live in the most impacted areas. Apart from creating therapies against novel variants, most research should focus on drug repurposing, which involves using medications that have previously been designed, tested for safety and efficacy, and are currently being used to treat another disease, COVID-19 [4,11].

In addition to medication repurposing, some dietary supplements may be helpful in the treatment of SARS-CoV-2 patients [68]. Malnutrition affects the immune system, including suppressing immunological responses and increasing virus susceptibility. As a result, enhancing gut health with a nutrient-dense diet will boost immunity against infections and illnesses [68]. Higher-than-recommended daily doses of minerals, including vitamins and zinc, may have a favorable effect, lowering the viral load and length of hospitalization in people with SARS-CoV-2. These micronutrients have been shown to have immunomodulatory properties and lessen the adverse effects of various diseases [69]. As a result, combining dietary techniques with other therapeutic regimens may be a safe and successful way to treat people infected with the Omicron strain.

Recently it has been advised that the public follow the COVID-19 guidelines as closely as possible and keep their vaccination doses up to date. Prior protection from natural illness, booster vaccinations, mandatory mask wear, and the deployment of effective preventative and control measures may help reduce the severity of the situation. Vaccinating the unvaccinated and immunocompromised people and delivering booster doses may also avert mortality and hospitalization [70]. To inhibit the spread of SARS-CoV-2 across the global population and generate novel SARS-CoV-2 variations, all governments should critically evaluate and address these concerns [71]. Animal models that are better appropriate for evaluating vaccination effectiveness and testing treatments against SARS-CoV-2 might be investigated. Understanding the immune response patterns following VOC infection is also suggested [71].

## 9 Conclusions and future directions

The Omicron version of SARS-CoV-2 has indicated that this virus can spread beyond the reach of currently available treatments. Future COVID-19 therapy should preferably have various characteristics such as solid efficacy in lowering the viral load and viral dissemination, broad-spectrum protection towards all VOCs, and an increased resistance threshold to prevent an exacerbating pursuit of the virus modifications in the future. Novel quick approaches for predicting the affinity and interactions between ACE2 and RBD, getting insights into the transmissibility and pathogenicity of new variations, and developing new diagnostic kits and vaccinations are required to supplement existing therapy and management options [72]. A significant factor in the emergence of omicron occurred in a country with a poor vaccine coverage rate. Undoubtedly, the emergence of this new variant highlights the critical importance of providing universal access to vaccination because allowing the virus to circulate in non-vaccinated populations, first freely, endangers such people with catastrophic COVID-19 incidence and mortality, and, second, enables the pathogen rapidly acquire genetic changes, which can significantly raise viral transmissibility and pathogenicity, or result in new devastating waves worldwide [70].

It's important to remember that the first two doses of the mRNA SARS-CoV-2 vaccine are less efficient in inducing neutralizing antibodies against the Omicron strain. Still, a third dose or breakthrough infection can help restore weak antibody responses [73]. Governments can embrace the strategy of providing booster doses of COVID-19 vaccines, especially to vulnerable communities such as immunocompromised people [74]. Additionally, international efforts must develop a vaccination program that employs a very effective vaccine to reach the most extensive possible coverage.

## Funding

No funding.

## Author contributions

Fahadul Islam: Conceptualization, Data curation, Writing-Original draft preparation, Writing- Reviewing and Editing. Manish Dhawan: Data curation, Writing-Original draft preparation, Writing- Reviewing and Editing. Mohamed. H. Nafady: Data curation, Writing-Original draft preparation, Writing- Reviewing and Editing. Talha Bin Emran: Conceptualization, Writing-Reviewing and Editing, Visualization. Saikat Mitra: Writing-Reviewing and Editing, Visualization. Om Prakash Choudhary: Writing-Reviewing and Editing, Visualization. Aklima Akter: Conceptualization, Supervision, Writing-Reviewing and Editing.

## Data availability statement

The data supporting this study's findings are available from the corresponding author upon reasonable request.

## Declaration of competing interest

The authors declare that they have no conflicts of interest.
